# The prognostic impact of microvascular invasion in patients with cholangiocarcinoma: A retrospective study

**DOI:** 10.1097/MD.0000000000049292

**Published:** 2026-06-26

**Authors:** Jungnam Lee, Seok Jeong, Don Haeng Lee, Jin-Seok Park

**Affiliations:** a Department of Internal Medicine, Inha University Hospital, Inha University School of Medicine, Incheon, South Korea; b Department of Internal Medicine, Shihwa Medical Center, Siheung, South Korea.

**Keywords:** cholangiocarcinoma, microvascular invasion, prognosis, surgical resection

## Abstract

Cholangiocarcinoma (CCA) is the second most prevalent primary liver cancer. Microvascular invasion (MVI) indicates more aggressive tumor behavior. While surgery is the primary curative treatment, recurrence, especially in the presence of MVI, remains a considerable concern. This study delves into the impact MVI has on the prognosis of CCA patients after surgical resection. This retrospective study was performed on 177 CCA patients who underwent surgical resection between January 2007 and December 2022. The primary study objective was to determine the impact of MVI on prognosis, especially in early-stage T1 and T2 cases. Clinicopathological findings, including tumor size, differentiation, and metastatic lymph node status, were meticulously analyzed. Of the 177 patients, 104 exhibited MVI (the MVI group), and 73 did not (the nMVI group). The MVI group had a higher incidence of metastatic lymph nodes (45.2% vs 19.2%, *P* < .01) and poorer tumor differentiation (*P* < .01). Median disease-free survival (DFS) and overall survival (OS) in the MVI group were notably shorter than in the nMVI group (*P* < .01 for both). In the T1 and T2 subgroups, the presence of MVI was consistently correlated with shorter DFS and OS. Multivariate analysis indicated that the presence of MVI, non-achievement of R0 resection, and higher baseline CA 19-9 levels were associated with poorer OS. This study shows that MVI plays a crucial role by negatively influencing the prognosis of CCA patients because its presence is significantly associated with reduced DFS and OS. These findings emphasize the need for vigilant postoperative monitoring and potential targeted treatments for CCA patients exhibiting MVI.

## 1. Introduction

Cholangiocarcinoma (CCA) is a lethal tumor of epithelial origin found throughout the biliary system or within liver tissue.^[1–4]^ CCA is the second most prevalent primary liver cancer, following hepatocellular carcinoma (HCC), and accounts for nearly 15% of all primary liver malignancies and 3% of all gastrointestinal tumors.^[5]^ Although CCA is relatively rare, its global incidence and death rates have increased noticeably in recent years. The worldwide incidence of CCA lies between 0.3 and 6 per 100,000 people annually.^[5]^ However, in Asian countries, including South Korea, its incidence is exceptional and exceeds 6 per 100,000 of the population per annum.^[6,7]^

Surgical resection remains the primary potentially curative treatment for CCA, but due to delayed diagnoses, resection rates are suboptimal. Furthermore, 5-year survival rates after surgery range between 25% and 50%,^[8–10]^ and even after achieving R0 resection, more than 50% of patients are confronted with the imminent risk of disease recurrence. Moreover, the risk of recurrence is particularly pronounced in patients with factors such as lymph node metastasis or poor tumor differentiation. Given these clinical complexities, contemporary guidelines strongly advocate adjuvant chemotherapy to enhance outcomes.^[11,12]^

Microvascular invasion (MVI) refers to the microscopic detection of tumor cells within vascular lumen.^[13]^ Studies have shown that in a background of HCC, MVI may be a form of occult micro-metastasis from a primary tumor, which can cause early recurrence after surgical resection and a poor prognosis.^[14,15]^ Lauwers et al reported that over half of HCC patients had MVI, which highlighted its status as a significant prognostic marker in HCC after surgery.^[16]^ In line with this, Chen et al, in a comprehensive analysis, concluded that MVI is an independent risk factor of poorer disease-free survival (DFS) and overall survival (OS) for solitary small HCCs post-hepatectomy.^[17]^ Furthermore, the prognostic implications of MVI in renal cell carcinoma have been substantiated in several studies.

Nevertheless, the precise impact of MVI on the clinical course of patients grappling with CCA has not been comprehensively investigated. Our previous studies revealed that MVI following CCA resection performed with curative intent is conspicuously correlated with adverse clinical outcomes. Building on this foundation, we undertook the present retrospective study with an enlarged cohort to ascertain the impact of MVI on the prognosis of CCA patients after surgical resection.

## 2. Methods

### 2.1. Study cohort

This retrospective study was conducted by reviewing the medical records of 246 patients, histologically diagnosed with CCA, who underwent surgical resection with curative intent at INHA University Hospital (Incheon, South Korea) between January 2007 and December 2022. The foundational principle for intrahepatic CCA surgery is to achieve hepatic resection with negative margins supplemented by regional lymphadenectomy of the porta hepatis. In cases of extrahepatic CCA, the primary objective remains complete resection with clear margins, accompanied by regional lymphadenectomy. Pathology specimens were reviewed to determine primary pathologic diagnoses and disease extents. Clinicopathological findings included tumor size, tumor differentiation and invasion, post-operative lymph node metastasis, and surgical margin statuses. Tumors were graded as poor, moderate, or well-differentiated. Final stages were recorded per the TNM classification system of the American Joint Committee On Cancer 8th Edition for CCA. Inclusion criteria were an age of ≥18 years, a diagnosis of CCA with confirmed pathologic staging, no history of previous chemotherapy or radiotherapy for another malignancy, and no evidence of synchronous cancer. Those that underwent other than R0 or R1 resection, with a different pathological type (e.g., neuroendocrine carcinoma or carcinosarcoma), and those that did not undergo an imaging examination within 6 months postoperatively were excluded. Sixty-nine patients were excluded according to these exclusion criteria, and thus, 177 CCA patients that underwent R0 or R1 resection were included in the analysis.

### 2.2. Clinical and pathologic information

During the 2 years following resection, patients underwent physical evaluations and blood tests, which included hematologic cancer markers (CA 19-9), at intervals of 3 to 6 months, and then biannually from years 2 to 5 years postoperatively. Diagnostic imaging, which included chest radiography, abdominal ultrasonography, computed tomography, and magnetic resonance imaging, was routinely conducted at a minimum biannually for 5 years postoperatively. After this period, imaging assessments were initiated based on clinical indications of potential relapse. Recurrence was determined based on radiological and/or cytological findings, and positron emission tomography-computed tomography was utilized in cases with ambiguous signs of recurrence. A diagnosis of tumor recurrence was made based on radiological or cytological verification independently of tumor marker statuses. After resection, patients underwent chemotherapy and/or radiotherapy as per guidelines, but treatments were individualized based on patient conditions and physicians’ clinical assessments.

### 2.3. Statistical interpretation

Disease-free survival (DFS) was defined as the time from surgery to the first manifestation of local or regional recurrence, and OS was defined as the time from surgical intervention to the patient’s passing or last recorded follow-up. The Kaplan–Meier technique was used to calculate survivals and the log-rank test to determine the significance of differences. Univariate Cox regression analysis was used to determine the hazard ratios associated with clinicopathological variables, and those variables found to be associated with DFS or OS by univariate analysis with *P*-values < .10 were entered into the multivariate analysis. The analysis was conducted using IBM SPSS software version 23.0 (IBM Corp., Armonk), and statistical significance was accepted for two-sided *P* values of < .05.

### 2.4. Ethics statement

The study protocol was approved by the Institutional Review Board of Inha University Hospital (INHAUH 2023-08-025), which waived the requirement for written informed consent due to the retrospective nature of the study. The study was conducted in accordance with the Declaration of Helsinki and Good Clinical Practice guidelines.

## 3. Results

### 3.1. Patient profiles and clinicopathological traits in the MVI and nMVI groups

**Figure 1.**
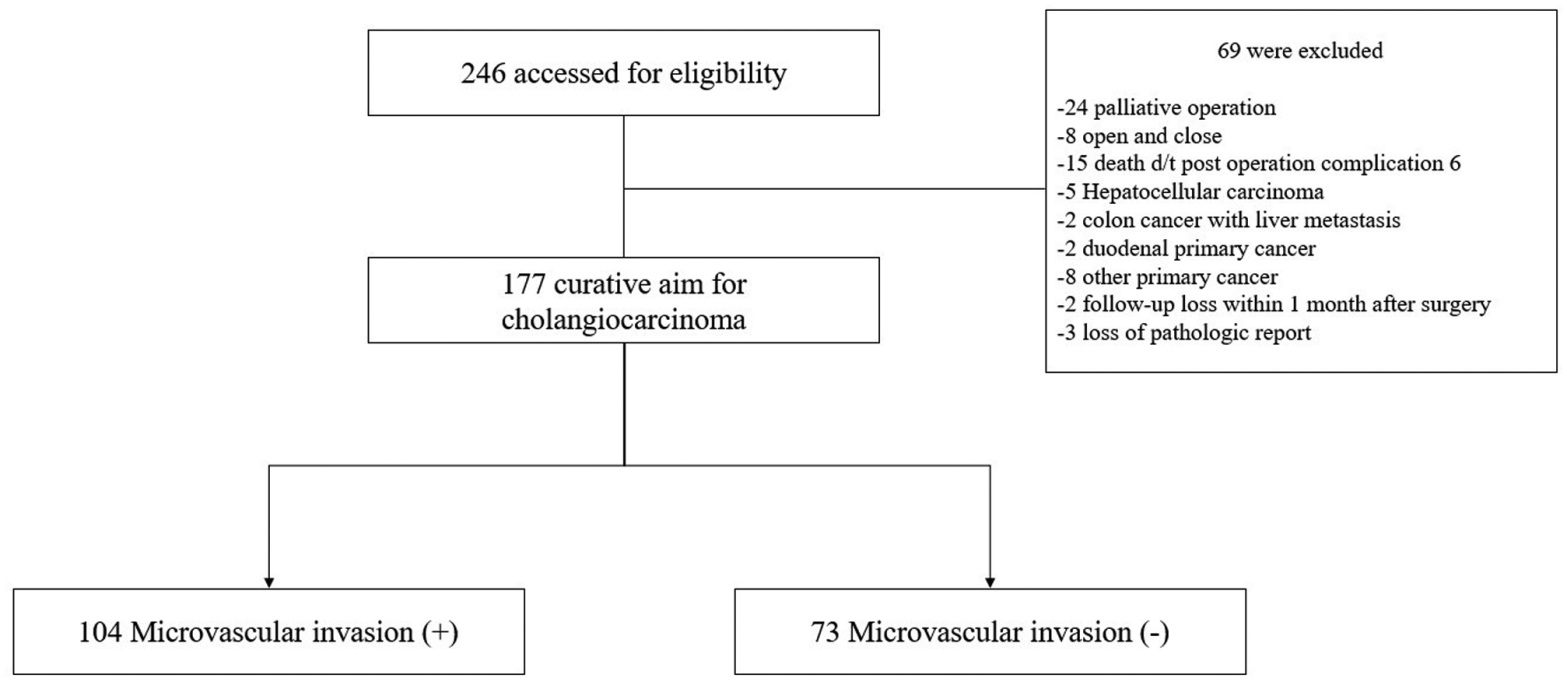
Flowchart of the patient selection process.

Of the 246 patients screened for this study, 69 were excluded, and the remaining 177 underwent surgery with a curative intent and were subsequently enrolled. The reasons for the 69 exclusions were as follows: 24 underwent a palliative operation, 8 received an open-and-close procedure, 15 succumbed to post-operative complications, 5 were diagnosed with HCC, 2 with colon cancer presenting with liver metastasis, 2 with primary duodenal cancer, and 8 with other primary cancers. In addition, 2 were lost to follow-up within a month after surgery, and 3 had missing pathologic reports (Fig. 1). Of the 177 study subjects, 104 (58.8%) were allocated to the MVI group and 73 (41.2%) to the nMVI group. Group baseline clinical characteristics are detailed in Table 1. Notably, the MVI group had a significantly higher metastatic lymph node rate (45.2% vs 19.2%, *P* < .01) and a greater prevalence of poorly differentiated tumors (*P* < .01). Furthermore, the rates of advanced American Joint Committee on Cancer T stages were significantly greater in the MVI group (T1 & T2: 69.2% vs 86.3%; T3 & T4: 30.8% vs 13.7%, *P* = .01), suggesting a potentially more aggressive disease course (Table 1).

**Table 1:** Baseline clinical characteristics of patients in the nMVI and MVI groups.

Variables	nMVI (n = 73)	MVI (n = 104)	P*-value
Age (yr)†	68 (45–90)	67 (35–85)	.85
Sex, male (%)	27	67	.54
CA 19-9, U/mL†	55.53 (0.9–9565.0)	94.73 (0.6–2400)	.51
AFP, ng/mL†	3.05 (0.5–14.2)	2.8 (0.6–360)	.42
Total bilirubin, mg/dL†	1.7 (0.2–20.9)	1.26 (0.1–24.2)	.43
AST, U/L†	78 (13.0–485.0)	52.5 (8.0–559.0)	.43
ALT, U/L†	77 (12–853)	63 (7.0–620.0)	.84
ALP, U/L†	239 (50–1234)	202 (37.0–2604)	.35
Histologic type (adenocarcinoma)			
Well-differentiated	26	12	<.01
Moderately differentiated	35	47	
Poorly differentiated	12	44	
Other	0	1	
R0 resection, n (%)	36 (49.3%)	38 (36.5%)	.09
Metastatic lymph node, n (%)	14 (19.2%)	47 (45.2%)	<.01
Perineural invasion, n (%)	39 (53.4%)	61 (58.7%)	.49
AJCC T stage, n (%)			
T1 & T2	63 (86.3%)	72 (69.2%)	.01
T3 & T4	10 (13.7%)	32 (30.8%)	
Adjuvant chemotherapy, n (%)	47 (64.4%)	84 (80.8%)	.01
Adjuvant radiotherapy, n (%)	12 (16.4%)	30 (28.8%)	.06

AFP = alpha-fetoprotein, AJCC = American Joint Committee on Cancer, ALP = alkaline phosphatase, ALT = alanine aminotransferase, AST = aspartate aminotransferase, CA 19-9 = carbohydrate antigen, MVI = microvascular invasion.

† Median (range).

* *P* values were calculated using the *t*-test for continuous variables and the Chi-square test for categorical variables.

### 3.2. Survival of patients stratified by microvascular invasion

**Figure 2.**
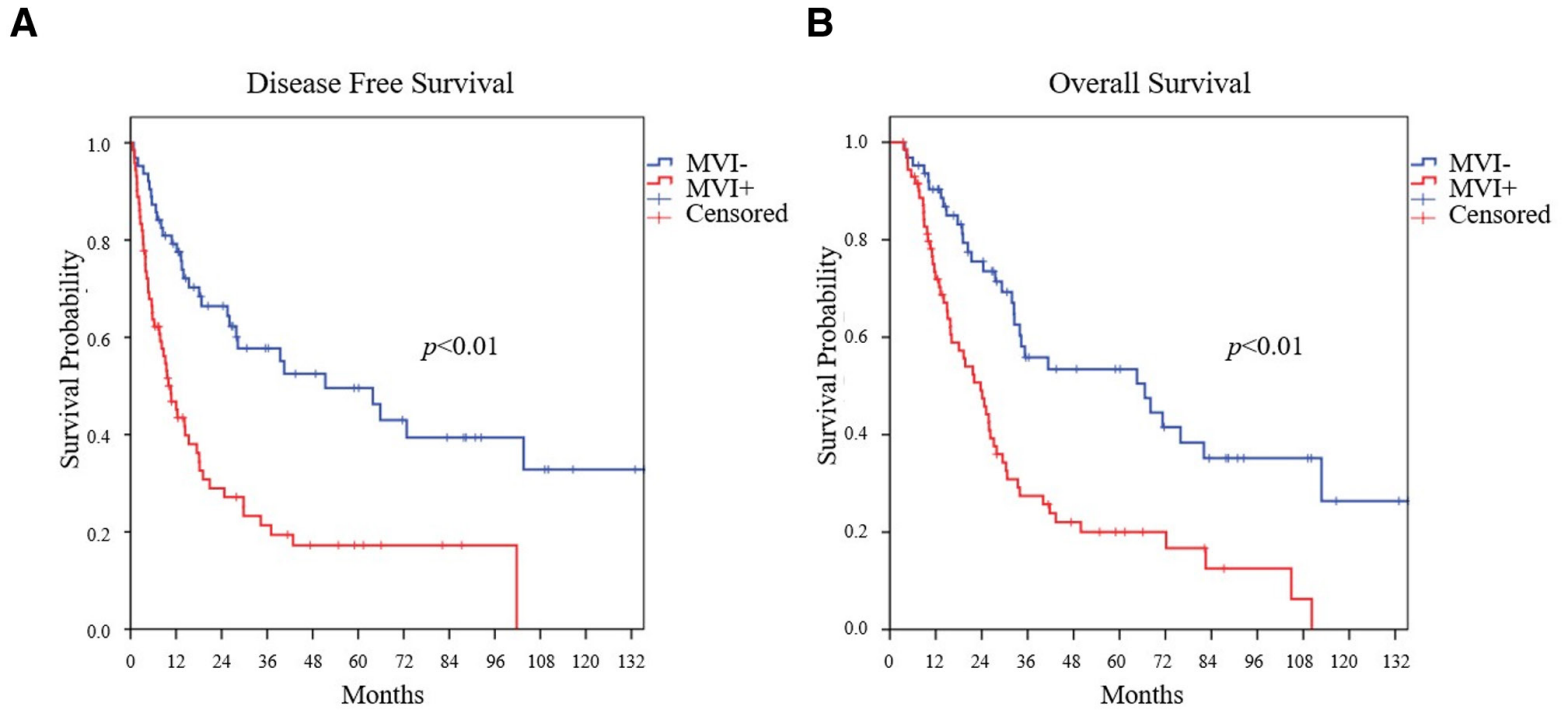
Kaplan–Meier survival curves for CCA patients stratified by MVI status: (A) DFS; (B) OS. CCA = cholangiocarcinoma, DFS = disease-free survival, MVI = microvascular invasion, OS = overall survival.

Results of the survival analysis performed on the MVI and nMVI groups are shown in Figure 2A and B. Median DFS and OS were 21.2 and 38.8 months, respectively, in the MVI group and 51.8 and 70.8 months, respectively, in the nMVI group. DFS rates for patients with or without MVI were 73.4% and 39.1%, respectively, at 1 year, and 56.0% and 17.4%, respectively, at 3 years; corresponding OS rates were 87.2 and 66.4% at 1 year and 54.0% and 24.6% at 3 years. Intergroup differences were significant for DFS and OS (both *P* < .01), meaning patients negative for MVI had better DFS and OS (Fig. 2A and B).

### 3.3. Survival of T1 and T2 patients stratified by microvascular invasion

**Figure 3.**
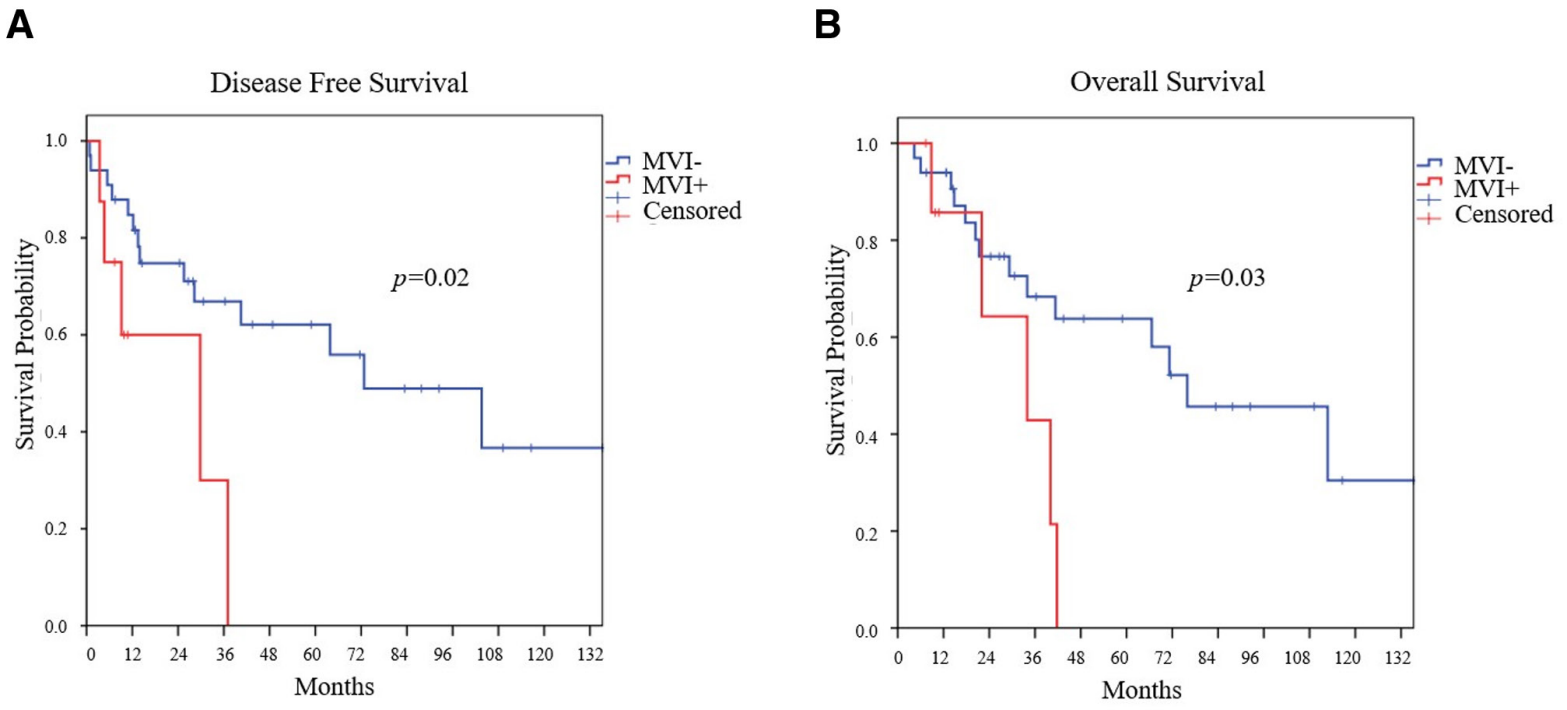
Kaplan–Meier survival curves for T1 CCA patients stratified by MVI status: (A) DFS; (B) OS. CCA = cholangiocarcinoma, DFS = disease-free survival, MVI = microvascular invasion, OS = overall survival.

**Figure 4.**
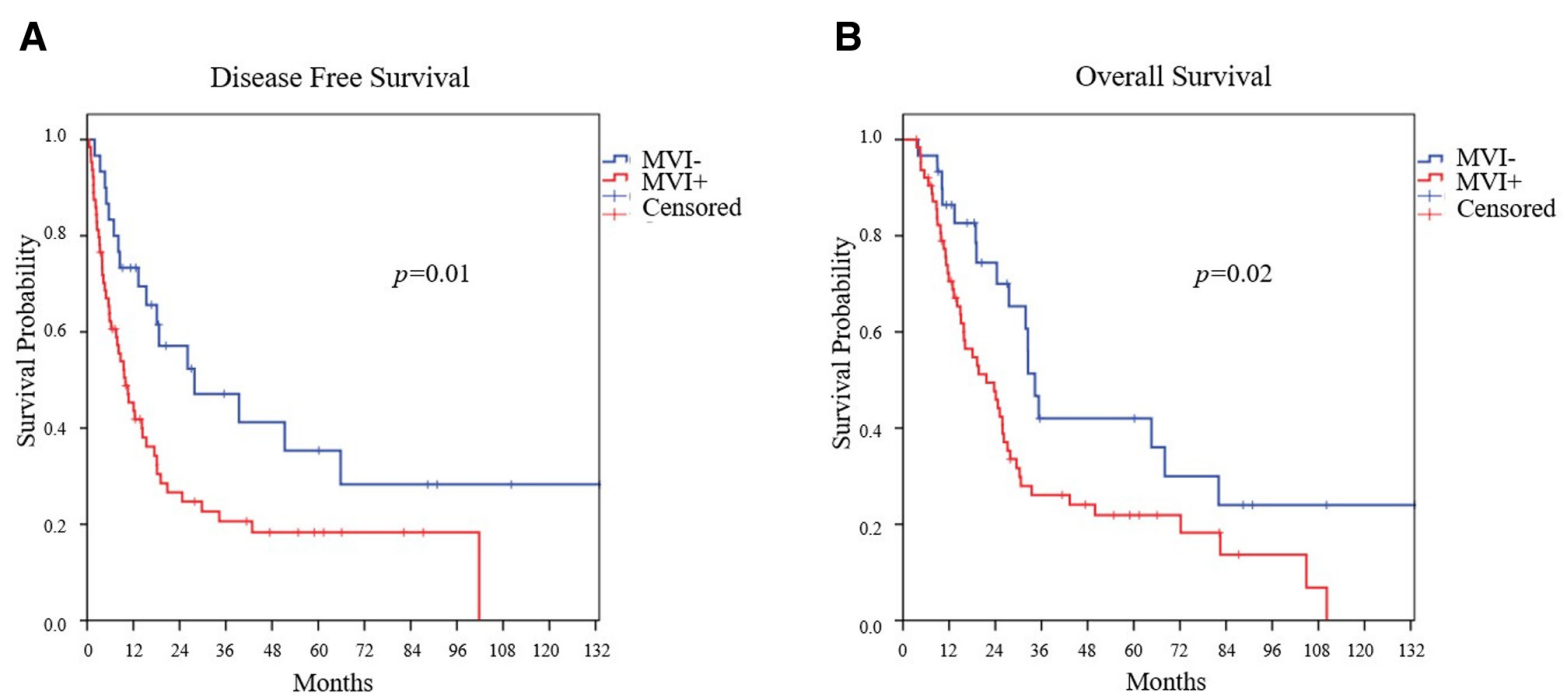
Kaplan–Meier survival curves for T2 CCA patients stratified by MVI status: (A) DFS; (B) OS. CCA = cholangiocarcinoma, DFS = disease-free survival, MVI = microvascular invasion, OS = overall survival.

Survival analysis results for T1 patients are shown in Figure 3A and B. Median DFS and OS for T1 patients with MVI were 75.6 and 86.1 months, respectively, but 184.4 and 192.5 months for T1 patients with nMVI. These differences were significant for DFS (*P* = .02) and OS (*P* = .03), indicating better DFS and OS for patients without MVI (Fig. 3A and B). Results for T2 patients are shown in Figure 4A and B. The median DFS and OS for T2 patients with MVI were 25.3 and 55.0 months, respectively, but 70.1 and 87.0 months for patients with nMVI, which was also significant for DFS (*P* = .01) and OS (*P* = .02), suggesting T2 patients without MVI have better prognoses for DFS and OS (Fig. 4A and B).

### 3.4. Univariate and multivariate analysis

Univariate and multivariate Cox proportional hazard regression analyses for DFS and OS were conducted on the 177 patients; results are presented in Table 2. Poor differentiation and the absence of MVI were associated with longer DFS by univariate and multivariate analyses. Regarding OS, univariate analysis revealed that poor tumor differentiation, achievement of R0 resection, absence of MVI, and lower baseline CA 19-9 levels were associated with longer OS. On the other hand, multivariate analysis showed longer OS was associated with the achievement of R0 resection, absence of MVI, and lower baseline CA 19-9 levels (Table 2).

**Table 2:** Univariate and multivariate Cox regression analysis results for the 177 patients.

Variables	Univariate analysis		Multivariate analysis	
	P*-value	HR (95% CI)	P*-value	HR (95% CI)
Univariate and multivariate Cox regression analyses for DFS.				
Histologic type	.04	1.64 (1.04–2.6)	.04	1.52 (1.02–2.27)
R0 resection	.40	0.82 (0.53–1.29)		
MVI	<.01	2.06 (1.26–3.38)	<.01	2.36 (1.55–3.58)
Metastatic lymph node	.08	1.58 (0.95–2.64)		
T stage	.56	1.18 (0.68–2.03)		
Adjuvant chemotherapy	.79	1.08 (0.63–1.85)		
Adjuvant radiotherapy	.20	1.36 (0.85–2.18)		
CA 19-9	.13	1.00 (1.00–1.00)		
Univariate and multivariate Cox regression analyses for OS				
Histologic type	.03	1.70 (1.04–2.77)	.13	1.44 (0.89–2.32)
R0 resection	.02	0.56 (0.33–0.92)	.01	0.52 (0.32–0.86)
MVI	.01	2.01 (1.18–3.43)	<.01	2.45 (1.48–4.05)
Metastatic lymph node	.33	1.33 (0.75–2.34)		
T stage	.05	1.79 (0.99–3.23)		
Adjuvant chemotherapy	.46	0.81 (0.47–1.42)		
Adjuvant radiotherapy	.38	1.24 (0.77–2.01)		
CA 19-9	.01	1.00 (1.00–1.00)	<.01	1.00 (1.00–1.00)

CA 19-9 = carbohydrate antigen, CI = confidence interval, DFS = disease-free survival, HR = hazard ratios, MVI = microvascular invasion.

* *P* values were calculated using the *t*-test for continuous variables and the Chi-square test for categorical variables.

## 4. Discussion

In this study, we comprehensively analyzed the association between MVI and prognosis in CCA after surgical resection with curative intent. Our retrospective evaluation revealed a significant correlation between the presence of MVI and shorter DFS and OS in CCA patients. Notably, even in our subgroup analysis focusing on early-stage T1 and T2 patients, those with MVI consistently exhibited significantly shorter DFS and OS. These findings underscore the critical role played by MVI in the prognosis of CCA and its potential implications for postoperative treatment decision-making.

MVI is considered a prominent prognostic indicator in HCC because it involves tumor dissemination via peritumoral blood vessels. Huang et al observed the prevalence of MVI increased with tumor progression.^[18]^ However, the influence of MVI appears to be dependent on stage. Specifically, in BCLC stage 0 (single tumor ≤ 2 cm) HCC, MVI was not correlated with DFS or OS, which is consistent with previous studies.^[18]^ In such cases, anatomical resection often effectively removes the tumor-bearing portal territory, and thus, eradicates micrometastatic nodules and MVI.^[19–22]^ Conversely, our research on CCA, focusing on T1 and T2 stages, unveiled a marked disparity in DFS and OS contingent on MVI status. To the best of our knowledge, this study is the first to analyze the impact of MVI on the prognosis of early-stage T1 and T2 CCA. Remarkably, even in T1 stage CCA, the presence of MVI signaled a compromised prognosis for DFS and OS. In addition, our study revealed that patients with MVI exhibited statistically significantly poorer differentiation and a higher incidence of metastatic lymph node positivity. Given these outcomes, MVI in CCA might serve as an even more potent prognostic factor than in HCC.

As regards the ongoing efforts to elucidate the mechanisms responsible for MVI in HCC, Zhao et al reported that the influence of MVI on disease progression can be attributed to the role of epithelial-mesenchymal transition.^[23]^ Epithelial-mesenchymal transition is a dynamic process wherein epithelial cells undergo transformation to acquire mesenchymal traits, which amplifies their invasive and metastatic capabilities.^[24]^ As this transition unfolds, cells relinquish their epithelial hallmarks, such as cell–cell adhesion and polarity, and embrace mesenchymal attributes, notably increased motility and resistance to apoptosis.^[25]^ This profound cellular shift paves the way for tumor cells to invade neighboring tissues and vascular structures.^[26]^ Such underlying mechanisms could explain the aggressive tendencies observed in CCA patients with MVI.^[23]^ Nonetheless, despite the insights provided by these findings, the mechanisms through which MVI impacts cancer progression remain to be elucidated, which underscores the need for further investigations.

Remarkably, in this study, MVI was observed in 58.8% of CCA patients. Considering the prevalence of concurrent mutations often found in CCA, there may be an association between MVI and mutations in fibroblast growth factor receptor (FGFR) and isocitrate dehydrogenase 1 (IDH1). FGFR2 mutations have been reported in 15% to 20% of biliary cancers, and IDH1 mutations in 15% to 20%.^[27]^ Furthermore, associations between FGFR and IDH1 mutations and angiogenesis and vascular invasion have been suggested in some cancers.^[27,28]^ As molecular analyses of CCA continue to advance, further investigations are warranted on associations between molecular anomalies and MVI. Recognizing the presence of MVI in CCA patients can profoundly influence treatment decisions and potentially guide clinicians toward more personalized therapeutic strategies. Furthermore, treatments targeting specific mutations or therapies that inhibit angiogenesis could provide substantial benefits, especially for patients with MVI.

While offering valuable insights, the present study also has several limitations. First, the retrospective design used is prone to selection bias and limits our ability to infer causality. Second, the data used were sourced from a single medical center and involved a single ethnic group, which potentially limits the generalizability of our findings. Third, the sample size was not sufficient for detailed subgroup analysis and may not have detected subtle differences. Fourth, our results have not been confirmed by external validation using an independent cohort, and though we made adjustments for known confounders, the potential for unmeasured or residual confounding remains. Finally, the absence of comprehensive genomic or molecular data for all patients means we might be missing further insights into the role of MVI in CCA prognosis.

In conclusion, our findings underscore the impact of MVI on the prognosis of CCA and reveal a marked disparity between the influence of MVI status on DFS and OS. This study is the first of its kind to analyze the impact of MVI on the prognosis of early-stage CCA, and we believe our findings have significant implications for post-operative strategies and future research directions.

## Author contributions

**Conceptualization:** Jin-Seok Park.

**Data curation:** Jungnam Lee.

**Formal analysis:** Jungnam Lee.

**Funding acquisition:** Jin-Seok Park.

**Investigation:** Jungnam Lee, Don Haeng Lee.

**Methodology:** Seok Jeong, Jin-Seok Park.

**Project administration:** Jin-Seok Park.

**Resources:** Seok Jeong, Jin-Seok Park.

**Software:** Seok Jeong.

**Validation:** Jungnam Lee, Don Haeng Lee.

**Visualization:** Jungnam Lee, Don Haeng Lee.

**Writing – original draft:** Jungnam Lee.

**Writing – review & editing:** Jin-Seok Park.
